# Transanal total mesorectal excision and transabdominal robotic surgery for rectal cancer: A retrospective study

**DOI:** 10.1016/j.amsu.2021.102902

**Published:** 2021-10-01

**Authors:** Hiroshi Oshio, Yukiko Oshima, Gen Yunome, Shinji Okazaki, Ichiro Kawamura, Yuya Ashitomi, Hiroaki Musha, Masaaki Kawai, Fuyuhiko Motoi

**Affiliations:** a Department of Surgery I, Yamagata University Hospital, 2-2-2 Iidanishi, Yamagata, Yamagata Prefecture, 990-9585, Japan; b Department of Surgery, Sendai Medical Center, 2-11-12 Miyagino, Miyagino-ku, Sendai, Miyagi Prefecture, 983-8520, Japan

**Keywords:** Robotic surgery, Laparoscopic surgery, Rectal cancer, Hybrid surgery, Transanal total mesorectal excision (TaTME)

## Abstract

**Background:**

Transabdominal robotic surgery and transanal total mesorectal excision (TaTME) are newly introduced strategies for rectal cancer. These procedures might have many advantages in rectal cancer treatment in terms of improving oncological and functional outcomes, especially in cases involving advanced cancer or technical difficulty. In the present study, we aimed to clarify the advantages and disadvantages of transabdominal robotic surgery and laparoscopic TaTME as a hybrid surgery for rectal cancer.

**Materials and methods:**

We retrospectively evaluated six patients who underwent hybrid surgery for rectal cancer from August 2018 to April 2020. Both clinical and pathological outcomes were assessed.

**Results:**

Two patients showed circumferential margin involvement both before and after neoadjuvant therapy. Three patients were planned to undergo hybrid surgery with intersphincteric resection because of a narrow pelvis. One patient was planned to undergo hybrid surgery for a giant tumor of >10 cm. The median length of hospitalization was 17 days. No patients required conversion to an open procedure. All patients underwent formation of defunctioning ileostomies. Two patients had a stapled anastomosis and four had a hand-sewn coloanal anastomosis. Complications included one case of anastomotic leakage, which was managed conservatively with ultrasound- and computed tomography-guided drainage and antibiotics. Histological analysis revealed that all specimens had a negative radial margin and distal margin. The median number of lymph nodes harvested was 17.5. Two patients showed extensive lymph node metastases, including lateral node metastasis.

**Conclusion:**

Hybrid surgery was performed safely and may improve oncological outcomes for rectal cancer. This technique has many potential benefits and would be alternative option in multimodal strategies for rectal cancer.

## Highlights

• No studies to date have focused on treatment of locally advanced rectal cancer in men using intersphincteric resection hybrid technology involving transabdominal robotic surgery with conventional transanal total mesorectal excision.
• We found that hybrid surgery is feasible and may improve the quality of total mesorectal excision and the rate of radical resection for advanced rectal cancer.
• Hybrid surgery may improve urinary and sexual function.
• This technique has many potential benefits and would be an alternative approach for rectal cancer.

## Abbreviations

TME: total mesorectal excision
TaTME: transanal total mesorectal excision
MRI: magnetic resonance imaging
DM: distal margin
CRM: circumferential resection margin
LPLND: lateral pelvic lymph node dissection
ISR: intersphincteric resection
RM: radial margin

## 1 Introduction

Rectal cancer is one of the most common malignant diseases worldwide [1]. Its prognosis has been improved by adjuvant and neoadjuvant chemotherapy; however, surgery is the mainstay of curative treatment. Total mesorectal excision (TME) is the standard of care for curative resection of rectal cancer. Notably, however, incomplete TME and/or positive resection margins increase the local recurrence rate [2]. Two large multicenter randomized clinical trials were unable to confirm the non-inferiority of laparoscopic surgery for rectal cancer compared with open surgery in terms of the pathological completeness of resected specimens [3,4]. Conversely, two major alternate trials reported evidence supporting the use of laparoscopic surgery in terms of pathological outcomes [5,6]. In recent studies involving magnetic resonance imaging (MRI) to evaluate the extent and completeness of TME through open abdominal and laparoscopic approaches, >30% of patients had a residual mesorectum [7,8]. These findings imply the need for improvement in the TME technique.

Transanal TME (TaTME) involves a “bottoms up” approach through transanal endoscopic platforms under magnification from both the abdominal and transanal fields [9,10]. This approach is superior in addressing the purse-string suture placement prior to starting the anal-side rectal dissection using insufflation and provides excellent direct vision compared with the abdominal approach [11]. These advantages facilitate meticulous dissection along the non-vascular TME plane to secure the distal margin (DM) and circumferential resection margin (CRM) [12]. Multiple prospective case series have revealed improved short-term oncologic safety such as low rates of CRM and DM positivity [13,14] and high rates of complete and near-complete TME specimen grades [14,15]. The technique may be an appealing option for challenging lower rectal tumors [[13], [14], [15]].

Robotic surgery uses articulating instruments that offer many technical advantages [16,17]. These features help to identify and preserve small anatomical structures, such as the pelvic plexus, and perform precise TME in the narrow pelvic space.

A hybrid procedure involving transabdominal robotic rectal surgery with TaTME would enhance oncological and functional outcomes, especially in cases involving oncological and technical difficulties. Few reports have focused on the use of such hybrid technology [18,19]. In this study, we retrospectively assessed the clinical and pathological outcomes of hybrid surgery with a focus on feasibility and efficacy. Although we would have liked to compare locally advanced tumors that showed circumferential margin involvement after neoadjuvant therapy, no such cases treated by robotic and laparoscopic surgery are reported in the literature. We also compared hybrid surgery versus transabdominal laparoscopic or robotic surgery with lateral pelvic lymph node dissection (LPLND) for lower rectal cancer.

## 2 Materials and Methods

This study was a subset analysis of “The safety and feasibility of robotically-assisted laparoscopic rectal cancer surgery using da Vinci Surgical System” (UMIN000019857, https://upload.umin.ac.jp/cgi-open-bin/ctr/ctr_view.cgi?recptno=R000022940) and focused on the feasibility and efficacy of hybrid surgery involving abdominal robotic rectal surgery with TaTME. This study has been reported in line with the STROCSS Criteria [20].

**Fig. 1:**
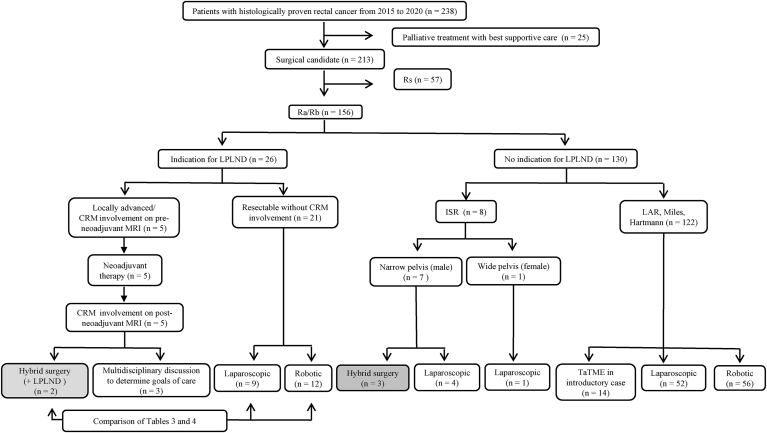
Flow chart of criteria for surgical treatment of rectal cancer. *Rs* rectosigmoid, *Ra* middle rectum, *Rb* lower rectum, *LPLND* lateral pelvic lymph node dissection, *CRM* circumferential resection margin, *MRI* magnetic resonance imaging, *ISR* intersphincteric resection, *LAR* low anterior resection, *TaTME* transanal total mesorectal excision.

We performed TME or total mesorectal specific excision for rectal cancer that was considered locally controllable by preoperative examination (Fig. 1). We only performed neoadjuvant treatment for clinical T4b locally advanced tumors that threatened the CRM on staging MRI. Additionally, rectal cancer surgery with LPLND was performed using laparoscopic or robotic surgery. Our indication criterion for LPLND was localization of the inferior tumor margin distal to the peritoneal reflection with extension beyond the muscularis propria [21]. In total, 210 patients underwent surgery for rectal cancer from 2015 to 2020. Conventional laparoscopic surgery was performed in 110 patients, robotic surgery in 100, TaTME in 14, hybrid surgery in 6, and LPLND in 23 (Fig. 1).

**Fig. 2:**
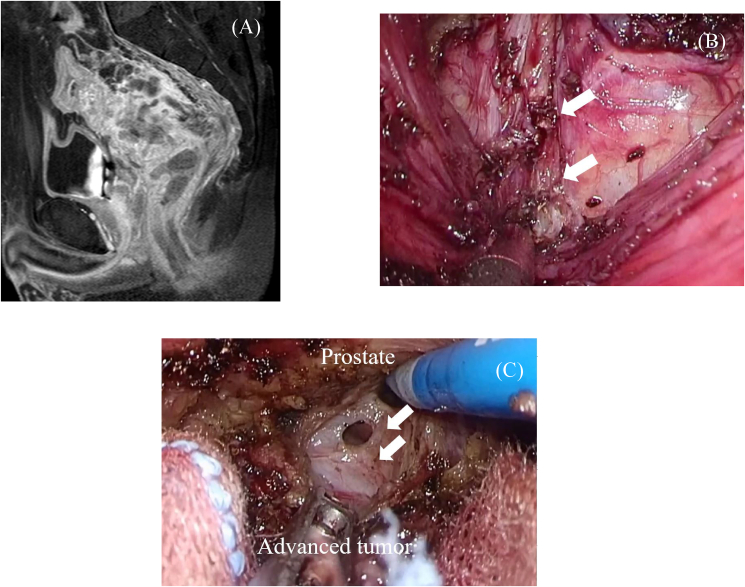
Preoperative and intraoperative findings in a patient who underwent hybrid surgery. (A) Preoperative magnetic resonance imaging after neoadjuvant treatment. (B) Dissection of the hiatal ligament (arrows) and below the endopelvic fascia of the distal tumor to secure the circumferential resection margin. (C) Dissection of Denonvilliers' fascia (arrows) to secure the circumferential resection margin.

Our treatment strategies for rectal cancer changed from 2015 to 2020: We first performed robotic surgery in October 2015, TaTME in March 2016, and hybrid surgery in August 2018. When we began performing hybrid surgery, we extended the treatment indication to include clinical T4b locally advanced tumors that threatened the CRM on staging MRI. Laparoscopic surgery was the main procedure until April 2018 because robotic surgery was not covered by public health insurance in Japan. Robotic surgery became the standard strategy after April 2018. TaTME was first performed in 14 cases of early cancer with a negative CRM on preoperative MRI as an introduction phase. Hybrid surgery was performed in six patients with rectal cancer, including three who required intersphincteric resection (ISR), one with a giant tumor of >10 cm, and two with clinical T4b locally advanced tumors that threatened the CRM on staging MRI. Two patients with locally advanced tumors showed CRM involvement both before and after neoadjuvant therapy. In these patients, we expected to encounter difficulty manipulating the pelvis because of the large tumor, narrow pelvis, and large uterine myoma. MRI showed invasion of the endopelvic fascia, and dissection below the endopelvic fascia of the distal tumor side was required to secure the CRM (Fig. 2 and Video 1) [22,23]. The TaTME approach was performed below the fascia, allowing optimal visualization and removal of the endopelvic fascia close to the distal tumor. Additionally, robotic surgery improves identification and preservation of small anatomical structures and facilitates precise TME and LPLND [24].

Supplementary video related to this article can be found at https://doi.org/10.1016/j.amsu.2021.102902

The following is/are the supplementary data related to this article:

Video 1 Surgical procedures in hybrid surgery. Video 1

All patients in this study underwent clinical examinations, total colonoscopy, abdominopelvic computed tomography, and pelvic MRI for preoperative staging. Patients with locally advanced cancer not amenable to curative surgery (clinical T4b) or suspected difficulty in securing the CRM received preoperative chemoradiotherapy and chemotherapy.

We used the tumor-node-metastasis classification, and the adequacy of the resection margins was evaluated using the radial margin (RM) rate [25]. Residual urine volume was measured as previously described [24].

### 2.1 Surgery

All six hybrid surgeries were performed by a single highly skilled and experienced surgeon using the da Vinci® Si™ Surgical System (Intuitive Surgical, Sunnyvale, CA, USA). The operating team was organized for a one-team TaTME procedure after the robotic surgery was complete. Robotic surgery was performed as previously described [24].

For TaTME, a Lone Star® retractor (Cooper Surgical, Trumbull, CT, USA) was used to retract the anal canal. The anal canal was then washed with a large amount of saline. A 4.0- × 5.5-cm GelPOINT® Path Transanal Access Platform (Applied Medical, Rancho Santa Margarita, CA, USA) was inserted into the anal canal. Three trocars were inserted in the platform in a triangular position^.^ A camera with a flexible tip (Olympus, Tokyo, Japan) was inserted through the superior trocar. Pneumorectum was established at 15 mmHg with an AirSeal® iFS Intelligent Flow System (CONMED, Utica, NY, USA). We prevented proximal rectal distension by inserting laparoscopic gauze into the rectal lumen during rectal insufflation. We used 2-0 Prolene suture to create a double purse-string to close the rectum distal to the lesion. This area was washed again with a large amount of saline. A planned circumferential mucosal mark was created at the edge of the circumferential radial mucosal folds. Full-thickness dissection was performed until the avascular perirectal plane was reached. Following circumferential identification of the endopelvic fascia, the extrafascial TME plane was identified and dissected posteriorly. The TaTME procedure was performed until cephalad dissection achieved a “rendezvous” to the abdominal dissected area [26,27].

Finally, we performed conventional laparoscopic anastomosis. In cases of low anterior resection, end-to-end anastomosis was performed using a standard single-stapling technique. The specimen was extracted through a 3- to 6-cm incision in the umbilical port. The anvil of the circular stapler was secured in place. We used 2-0 Prolene suture to create a purse-string to 12 stitches on the edge of the rectal dissection and inserted the circular stapler from there. We usually used a PROXIMATE® Intraluminal Stapler (Ethicon Endo-Surgery, Cincinnati, OH, USA) ^or an^ EEA™ circular stapler with SST Series™ technology (25-mm and 4.8-mm staples; Medtronic, Minneapolis, MN, USA). In cases of ISR, we performed transanal intersphincteric dissection and coloanal hand-sewn anastomosis.

### 2.2 Statistics

Continuous data were compared with the paired *t*-test, and categorical data were compared with the chi-squared test and Fisher's exact test. Statistical analyses were performed using JMP (SAS Institute Inc., Cary, NC, USA) and R (http://www.R-project.org/). A p value of <0.05 was considered statistically significant.

## 3 Results

From September 2018 to April 2020, six patients (five men, one woman) aged 38–76 years (mean, 60.3 years) underwent hybrid surgery for rectal lesions. Table 1 shows their characteristics and short-term outcomes.

**Table 1:** Characteristics and short-term outcomes of patients who underwent hybrid surgery.

Clinical and surgical characteristics						
Patient	1	2	3	4	5	6
Age (years)	38	60	76	60	73	55
Sex	Male	Female	Female	Male	Male	Male
BMI (kg/m^2^)	21.6	19.3	25.7	18.7	23.8	26.9
ASA-PS	2	1	2	3	2	1
Surgery	LAR	SLAR	SLAR	ISR	ISR	ISR
LPLND (+)	Yes	Yes	No	No	No	No
Covering stoma	Yes	Yes	Yes	Yes	Yes	Yes
Stoma	No	No	No	No	No	No
Anastomosis	Stapled	Stapled	Hand-sewn	Hand-sewn	Hand-sewn	Hand-sewn
**Tumor and histological characteristics**						
Patient	1	2	3	4	5	6
Tumor location (cm)^a^	7	6	5	4	3	4
Tumor diameter (mm)	60	25	235	45	23	11
Final tumor pathology	Adenocarcinoma	Adenocarcinoma	High-grade tubulovillous adenoma	Adenocarcinoma	Adenocarcinoma	Adenocarcinoma
cTNM (JSCCR) ypTNM	T4bN3M0 T3N3M0	T4bN3M1a (liver) T4bN3M1a (liver)	–	T3N0M0	T1bN0M0	T1bN0M0
Neoadjuvant chemotherapy	Yes	Yes	No	No	No	No
Neoadjuvant chemoradiotherapy	Yes	No	No	No	No	No
CRM involvement on post-neoadjuvant MRI	Yes	Yes	No	No	No	No
RM (+)	No	No	No	No	No	No
Proximal margin (mm)	230	160		165	110	180
Distal margin (mm)	50	20		10	5	15
TME grade	Complete	Complete	Complete	Complete	Complete	Complete
Number of lymph nodes harvested	32	21	15	25	7	5
Number of positive lymph nodes	14	3	0	0	0	0
**Short-term outcomes**						
Patient	1	2	3	4	5	6
Conversion to open surgery	No	No	No	No	No	No
Operation time (min)	846	582	673	703	520	406
Intraoperative complications	No	No	No	No	No	No
Blood loss (mL)	60	50	200	30	95	75
Hospital stay (days)	43	18	13	24	14	16
Complications	Anastomotic leakage	Urinary retention	No	Ischemic colitis	No	No
Urinary retention (>50 mL)	No	Yes	No	No	No	No

*BMI* body mass index, *ASA-PS* American Society of Anesthesiologists physical status, *LAR* low anterior resection, *SLAR* super-low anterior resection, *ISR* intersphincteric resection, *LPLND* lateral pelvic lymph node dissection, *TNM* tumor, node, metastasis, *JSCCR* Japan Society for Cancer of the Colon and Rectum, *CRM* circumferential resection margin, *MRI* magnetic resonance imaging, *RM* radial margin, *TME* total mesorectal excision.

a Distance from anal verge.

Three patients underwent low or super-low anterior resection, and three underwent ISR. Two patients underwent LPLND. A covering ileostomy was created in all patients. Two patients had a stapled anastomosis and four had a hand-sewn coloanal anastomosis (Table 1).

Adenocarcinoma was confirmed histologically in five patients. One patient had liver metastases. The mean distance from the anal verge was 4.8 cm (range, 3–7 cm). Two patients had suspected CRM involvement before and after neoadjuvant treatment. Histological analysis showed that all specimens had a negative RM and DM (5–50 mm). The median number of lymph nodes harvested was 17.5 (5–32). Two patients had a high lymph node burden of disease (3–14 positive lymph nodes and 1 lateral lymph node metastasis in each patient). These patients had been preoperatively diagnosed with N3 cancer on MRI and underwent robotic LPLND (Table 1).

The median hospital stay was 17 days (range, 13–43 days). No intraoperative complications occurred, and no patients required conversion to an open procedure. Complications included one case of anastomotic leakage, which was managed conservatively with ultrasound- and computed tomography-guided drainage and antibiotics (Table 1).

We compared hybrid surgery versus transabdominal laparoscopic or robotic surgery with LPLND for lower rectal cancer. Table 2 shows the background characteristics of the patients treated with hybrid, laparoscopic, and robotic surgery. The proportions of patients with advanced rectal cancer with metastasis to lateral lymph nodes (N3) and distant metastasis (M1a), neoadjuvant chemotherapy, and CRM involvement on post-neoadjuvant MRI were significantly higher in the hybrid surgery group than in the laparoscopic and robotic surgery groups. All patients underwent LPLND (Table 3).

**Table 2:** Clinical characteristics (hybrid vs. laparoscopic vs. robotic surgery).

	I: Hybrid	II: Laparoscopic	III: Robotic	p (I vs. II)	p (I vs. III)
Number of patients	2	9	12		
Median age, years	49.0	59.7	58.6	0.246	0.144
Male sex	1 (50.0%)	7 (77.8%)	9 (75.0%)	0.425	0.649
Median BMI (kg/m^2^)	20.5	23.2	22.0	0.248	0.495
Location					
Rs	0 (0.0%)	0 (0.0%)	0 (0.0%)		
Ra	1 (50.0%)	0 (0.0%)	0 (0.0%)		
Rb	1 (50.0%)	9 (100%)	12 (100%)		
Median size (mm)	42.5	55.3	52.3	0.243	0.412
T				0.391	0.277
Is	0 (0.0%)	0 (0.0%)	0 (0.0%)		
1	0 (0.0%)	0 (0.0%)	0 (0.0%)		
2	0 (0.0%)	2 (22.2%)	2 (16.7%)		
3	1 (50.0%)	6 (66.7%)	9 (75.0%)		
4	1 (50.0%)	1 (11.1%)	1 (8.3%)		
N				0.012	0.003
0	0 (0.0%)	0 (0.0%)	4 (33.3%)		
1	0 (0.0%)	7 (77.8%)	4 (33.3%)		
2	0 (0.0%)	2 (22.2%)	2 (16.7%)		
3	2 (100%)	0 (0.0%)	1 (8.3%)		
M (+)	1 (50.0%)	0 (0.0%)	0 (0.0%)	0.026	0.119
ASA-PS					
1	1 (50.0%)	3 (33.3%)	6 (50.0%)		
2	1 (50.0%)	6 (66.7%)	5 (41.7%)		
3	0 (0.0%)	0 (0.0%)	1 (8.3%)		
4	0 (0.0%)	0 (0.0%)	0 (0.0%)		
Neoadjuvant chemotherapy	2 (100%)	0 (0.0%)	1 (8.3%)	0.0009	0.003
CRM involvement on post-neoadjuvant MRI	2 (100%)	0 (0.0%)	0 (0.0%)	0.0009	0.0002

*Hybrid* hybrid surgery with transanal total mesorectal excision and transabdominal robotic surgery, *Laparoscopi*c pure transabdominal laparoscopic surgery, *Robotic* pure transabdominal robotic surgery, *BMI* body mass index, *Rs* rectosigmoid, *Ra* middle rectum, *Rb* lower rectum, *ASA-PS* American Society of Anesthesiologists physical status, *CRM* circumferential resection margin, *MRI* magnetic resonance imaging.

Underlined text indicates a statistically significant difference.

**Table 3:** Surgical characteristics (hybrid vs. laparoscopic vs. robotic surgery).

	I: Hybrid	II: Laparoscopic	III: Robotic	p (I vs. II)	p (I vs. III)
Number of patients	2	9	12		
Procedure				0.338	0.533
HAR	0 (0.0%)	0 (0.0%)	0 (0.0%)		
LAR	2 (100%)	6 (66.7%)	10 (83.3%)		
ISR	0 (0.0%)	0 (0.0%)	0 (0.0%)		
Hartmann	0 (0.0%)	0 (0.0%)	0 (0.0%)		
Miles	0 (0.0%)	3 (33.3%)	2 (16.7%)		
LPLND (+)	2 (100%)	9 (100%)	12 (100%)	1.000	1.000
Covering stoma	2 (100%)	5 (83.3%)	10 (83.3%)	0.537	0.371
Permanent stoma	0 (0.0%)	3 (33.3%)	2 (16.7%)	–	–
Median number of lymph nodes harvested	26.5 (21–32)	34.1 (14–75)	44.8 (13–76)	0.583	0.215

*Hybrid* hybrid surgery with transanal total mesorectal excision and transabdominal robotic surgery, *Laparoscopic* pure transabdominal laparoscopic surgery, *Robotic* pure transabdominal robotic surgery, *HAR* high anterior resection, *LAR* low anterior resection, *ISR* intersphincteric resection, *LPLND* lateral pelvic lymph node dissection.

The mean surgical duration was significantly longer in the hybrid than laparoscopic and robotic groups. The estimated blood loss volume was significantly lower in the robotic than hybrid group (Table 4).

**Table 4:** Short-term outcomes (hybrid vs. laparoscopic vs. robotic surgery).

	I: Hybrid	II: Laparoscopic	III: Robotic	p (I vs. II)	p (I vs. III)
Number of patients	2	9	12		
Conversion	0	0	0	1.000	1.000
Median operation time (min)	645.6 (582–711)	475.0 (329–618)	466.8 (399–595)	0.036	0.002
Median blood loss (mL)	55.0 (50–60)	33.3 (15–70)	13 (1–30)	0.811	0.0002
Median hospital stay (days)	30.5	20.7	17.7	0.096	0.031
RM (+)	0 (100%)	0 (0.0%)	1 (8.3%)	1.000	0.672
Median distal margin (mm)	36.0 (22–50)	29.2 (8–58)	24.6 (10–40)	0.742	0.194
Complications	2 (100%)	3 (33.3%)	4 (25.0%)	0.089	0.049
SSI	0 (0.0%)	1 (11.1%)	2 (8.33%)	0.621	0.533
Ileus	0 (0.0%)	0 (0.0%)	1 (8.33%)	–	0.571
Anastomotic leakage	1 (50.0%)	0 (0.0%)	0 (0.0%)	0.026	0.035
Urinary retention (>50 mL)	1 (50.0%)	1 (11.1%)	1 (8.33%)	0.197	0.119
Bleeding	0 (0.0%)	1 (11.1%)	0 (0.0%)	0.621	–

*Hybrid* hybrid surgery with transanal total mesorectal excision and transabdominal robotic surgery, *Laparoscopic* pure transabdominal laparoscopic surgery, *Robotic* pure transabdominal robotic surgery, *RM* radial margin, *SSI* surgical site infection.

Underlined text indicates a statistically significant difference.

No patients in either group required conversion. The overall morbidity rate was lower in the robotic than hybrid group. No patient in the hybrid or laparoscopic group had a positive RM. The hospital stay was significantly longer in the hybrid than robotic group (Table 4). In the comparison of hybrid surgery and transabdominal laparoscopic or robotic surgery for men undergoing ISR, no significant differences in short-term outcomes were found (data not shown).

## 4 Discussion

Robotic transabdominal surgery is expected to overcome some of the limitations of laparoscopic surgery for rectal cancer [16,17]. Several non-randomized studies have suggested that robotic surgery may improve patients’ quality of life by preserving urinary and sexual function [24,[28], [29], [30]]. Additionally, chemoradiotherapy is not widely used in Japan. Reports from Japan show an overall survival improvement with LPLND [31], and recent reports have shown a benefit with LPLND after chemoradiotherapy [32]. Although LPLND is technically difficult in areas close to large vessels and nerves in the narrow and complex pelvic region, robotic surgery is expected to improve the quality and technical difficulty of LPLND [24,33,34]. Moreover, robotic surgery allows many experienced surgeons to more easily approach the pelvic floor. However, even robotic surgery often has limited access to the deep pelvic floor near the tumor in patients with a narrow pelvis, obesity, a bulky mesorectum, advanced or recurrent tumors, large tumors, or a large prostate or uterus. Additionally, the ROLLAR trial (a randomized controlled trial of robotic-assisted versus conventional laparoscopic surgery for rectal cancer) showed that the distal incision and circumferential dissection of TME remains controversial regardless of whether the transabdominal approach is robotic or laparoscopic [35]. TaTME simplifies TME dissection by ensuring an appropriate DM of the cancer while simultaneously providing a technique for manipulating the distal one-third of the highest-risk area in terms of CRM and DM positivity during TME dissection [36]. Therefore, in these cases of advanced rectal tumors, a hybrid of two approaches would provide many benefits by combining the advantages of abdominal robotic surgery and TaTME. The results of the present study have demonstrated that hybrid surgery provides acceptable short-term oncological outcomes. The ability to perform curative surgery is particularly valuable for patients with severe locally advanced cancer that threatens the CRM on staging MRI and lateral lymph node metastasis.

An educational program to acquire the skills necessary for TaTME is an important prerequisite. Concerns about the relationship between the surgical technique and recurrence highlight the need for a standardized educational program. According to some reports, 40 to 51 cases are required to complete the learning curve of TaTME [37,38]. The incidence of local recurrence is significantly higher in low-volume than high-volume TaTME centers (8.9% vs. 2.8%, respectively) [39]. Efforts should be directed toward the implementation of mechanisms that mitigate the risk of negative patient outcomes during skill acquisition by surgeons [40]. We introduce TaTME after sufficient education through cadaver training, knowledge of procedural pitfalls, and invitation of a surgical proctor according to strict introduction standards [[41], [42], [43]]. If the precise anatomical plane is not clear in the introductory phase, we do not proceed with TaTME and instead use the abdominal approach to reveal the anatomy.

D'Andrea et al. [44] reported that the indications for TaTME performed by surgeons beyond the learning curve were expanded to include patients with more advanced tumors involving a history of major abdominal or pelvic surgery, local recurrence, or cT4/cT3 tumors that threaten the CRM or internal anal sphincter. Despite the inclusion of more advanced tumors, the short-term oncologic outcomes in this report are reasonable, and the rate of CRM positivity was 3.9% despite a predicted rate of 25.5% based on staging MRI [44]. We have performed TaTME in about 30 patients to date. During TaTME for locally advanced rectal cancer, we carefully proceed with the distal end incision and try to avoid forceful dissection. We have not experienced RM positivity in any cases of TaTME. In Japan, the adequacy of resection margins is evaluated using the RM rate [45]. Fig. 1 shows that before the introduction of hybrid surgery, surgical treatment was not performed in cases of CRM involvement on post-neoadjuvant MRI. After the introduction of hybrid surgery in August 2018, CRM involvement on post-neoadjuvant MRI was treated surgically. We believe that hybrid surgery may achieve CRM negativity in these cases. Since the introduction of hybrid surgery in August 2018, the RM-positive rate has decreased from 5.4% (before its introduction) to 3.1% (after its introduction) (p = 0.4795) despite the fact that suspected CRM involvement is an indication for surgery.

We also apply hybrid surgery to ISR in male patients, in whom a narrow pelvis and obesity are risk factors for anastomotic leakage after ISR [46]. Males have a longer anal canal than females [47], which may make surgery technically difficult to perform. Surgeons prefer tools that offer fine surgical dexterity and stable optical magnification, especially in cases involving rectal dissection at the level of the anal sphincter for low rectal cancer.

The long duration of hybrid surgery in patients with T4b cancer can be explained by the high proportion of these patients who underwent preoperative chemotherapy (100% in hybrid surgery and 0% in robotic and laparoscopic surgery). Because preoperative treatment is indicated for patients with severely advanced cancer undergoing hybrid surgery, it is considered natural to extend the operation time. The duration might be shortened by using a two-team procedure in which robotic surgery and TaTME are performed simultaneously. The median hospital stay for patients who underwent hybrid surgery was 17 days (range, 13–43 days). With the exception of the patient who developed anastomotic failure and required hospitalization for 44 days, there was no significant difference between hybrid surgery and robotic/laparoscopic surgery. The extension of the postoperative hospital stay may have been related to the need to adjust to creation of the ostomy pouch in our institution. The complication rate was lower in the robotic than hybrid surgery group. We believe that this is acceptable because hybrid surgery is more often indicated for advanced cancers. Most notably, we were able to secure the CRM in cases of advanced rectal cancer with suspicion of CRM involvement on preoperative MRI.

In summary, we have demonstrated that hybrid surgery may allow improved operative vision and serve as a feasible option for oncologically safe rectal dissection along the TME plane. This procedure has two major advantages. First, the robotic approach facilitates identification and preservation of small anatomical structures, such as the pelvic plexus; allows for precise total TME in the narrow pelvic space; and improves the operability of LPLND [24]. Patients’ urinary and sexual function disorders have been shown to be more greatly improved after robotic than laparoscopic surgery [24]. Second, TaTME allows the surgeon to confidently achieve a DM under direct visualization and choose whether to take wider margins such as above or below the endopelvic fascia when there are concerns about CRM positivity [22,23]. We believe that the possibility of a secure CRM will increase by properly using the dissection layer from multiple directions. Based on these advantages, hybrid technology for abdominal robotic rectal surgery with TaTME might be indicated for patients with giant tumors, bulky tumors, a narrow pelvis, obesity, and neoadjuvant chemoradiotherapy as well as male patients undergoing ISR. Hybrid surgery has advantages for patients with a narrow pelvis and complex tumors because of potential involvement of the endopelvic fascia and difficulty manipulating the pelvis. Our results suggest that hybrid surgery might improve curative resection rates after complex rectal cancer surgery.

This study has several limitations. First, this was a retrospective study involving a small number of patients. Second, urinary and sexual function was not evaluated. Patients undergoing adjuvant chemotherapy for advanced cancer often do not answer these questionnaires. Third, long-term oncological results were not evaluated. The incidence of local recurrence, progression-free survival, overall survival, and urinary and sexual function should be evaluated to assess the true advantages of hybrid surgery for rectal cancer.

## 5 Conclusion

Hybrid surgery was safely performed and showed the possibility to improve oncological outcomes for advanced rectal cancer. This technique has many potential benefits and will be a potential option for rectal cancer treatment.

## Availability of data and material

Data can be made available on request.

## Ethics approval

The study protocol was approved by the local ethics committees of Sendai Medical Center (reference number: 27-8). The research was conducted in accordance with the 1964 Declaration of Helsinki and its later amendments.

## Funding

This research was self-funded and no additional funding was provided by third parties.

## Author contribution

HO described and designed this manuscript. YO, GY were the surgeon who did the operation and contributed to the critical revision of this manuscript. SO, IK, HM, MK contributed to the critical revision of this manuscript. FM contributed to the critical revision and final approval of this manuscript.

## Research registration

This study was registered with Research Registry (UMIN reference no. UMIN000019857, https://upload.umin.ac.jp/cgi-open-bin/ctr/ctr_view.cgi?recptno=R000022940).

## Guarantor

Hiroshi Oshio.

Fuyuhiko Motoi.

## Patient consent

Informed consent was obtained from all patients.

## Provenance and peer review

Not commissioned, externally peer-reviewed.

## Declaration of competing interest

None.
